# Integrated analysis of single-cell RNA-seq and spatial transcriptomics to identify the lactylation-related protein TUBB2A as a potential biomarker for glioblastoma in cancer cells by machine learning

**DOI:** 10.3389/fimmu.2025.1601533

**Published:** 2025-06-26

**Authors:** Yifan Xu, Chonghui Zhang, Jinpeng Wu, Pin Guo, Nan Jiang, Chao Wang, Yugong Feng

**Affiliations:** Department of Neurosurgery, The Affiliated Hospital of Qingdao University, Qingdao, China

**Keywords:** lactylation, glioblastoma, single-cell RNA sequencing, spatial transcriptomics, IQR

## Abstract

**Background:**

An increasing number of studies have revealed a link between lactylation and tumor initiation and progression. However, the specific impact of lactylation on inter-patient heterogeneity and recurrence in glioblastoma (GBM) remains to be further elucidated.

**Methods:**

We employed functional enrichment algorithms, including AUCell and UCell, to assess lactylation activity in GBM cancer cells. Additionally, we introduced the interquartile range (IQR) method based on a set of lactylation-related genes (LRGs) to reevaluate the extent of lactylation production within the cancer population at the single-cell resolution. By reconstructing the spatial transcriptomics of hematoxylin and eosin (HE)-stained sections, we further evaluated the lactylation activity in GBM tissues. Subsequently, We employed machine learning algorithms to identify hub genes significantly associated with elevated lactylation levels in GBM. Finally, we experimentally validated the emulsification efficiency and quantified the expression levels of hub genes in human GBM samples.

**Results:**

Our study innovatively demonstrated a markedly elevated global lactylation level in GBM and validated it as an independent prognostic factor for GBM. We established a prognostic gene model associated with emulsification in GBM. Furthermore, the machine learning-based model identified SSBP1, RPA3 and TUBB2A as potential biomarkers for GBM. Notably, the expression levels of these three hub genes and the lactylation level of TUBB2A in GBM tissues were significantly higher compared to those in normal tissues.

**Conclusions:**

We propose and validate a IQR lactylation screening method that provides potential insights for GBM therapy and an effective framework for developing gene screening models applicable to other diseases and pathogenic mechanisms.

## Introduction

GBM is a highly aggressive form of brain tumor that belongs to the glioblastoma subtype originating primarily in glial cells, which are support cells in the brain (1). It is one of the most prevalent and lethal primary brain tumors among adults (2). Based on molecular and genomic characteristics, GBM can be categorized into distinct subtypes, including IDH mutants and wild types, with significant implications for prognosis and treatment strategies (3, 4). Due to its intricate nature, studying GBM holds immense significance in oncology as it drives the advancement of novel therapeutic approaches such as immunotherapy, targeted therapies, and gene therapy (5, 6).

Lactylation refers to the process in which lactic acid molecules form ester or covalent bonds with proteins or other biomolecules, thereby modifying protein function, stability, and interaction (7). The significance of lactylation in cellular metabolism has gained increasing attention, particularly within the tumor microenvironment. Cancer cells often rely on aerobic glycolysis (8) for energy production, leading to an accumulation of lactic acid (7, 9). Lactylation, as a post-translational modification, is believed to exert significant regulatory effects on signal transduction and metabolic reprogramming in GBM cells (10). Research has demonstrated that lactylation of specific key proteins can modulate their functionality, including the regulation of cell cycle progression, apoptosis, and metabolic pathways (11, 12). The GBM is characterized by its heightened metabolic activity and aberrant angiogenesis (13, 14). Cancer cells satisfy their demand for rapid proliferation through upregulation of glycolysis and subsequent lactic acid production (15). Additionally, the accumulation of lactic acid results in tumor microenvironment acidification, which not only impacts Cancer cell proliferation but also potentially hinders immune cell function and facilitates immune evasion by tumors (16). Moreover, the acidic environment can influence the functionality of tumor-associated fibroblasts and vascular endothelial cells, thereby further promoting tumor growth and metastasis (17, 18).

**Figure 1:**
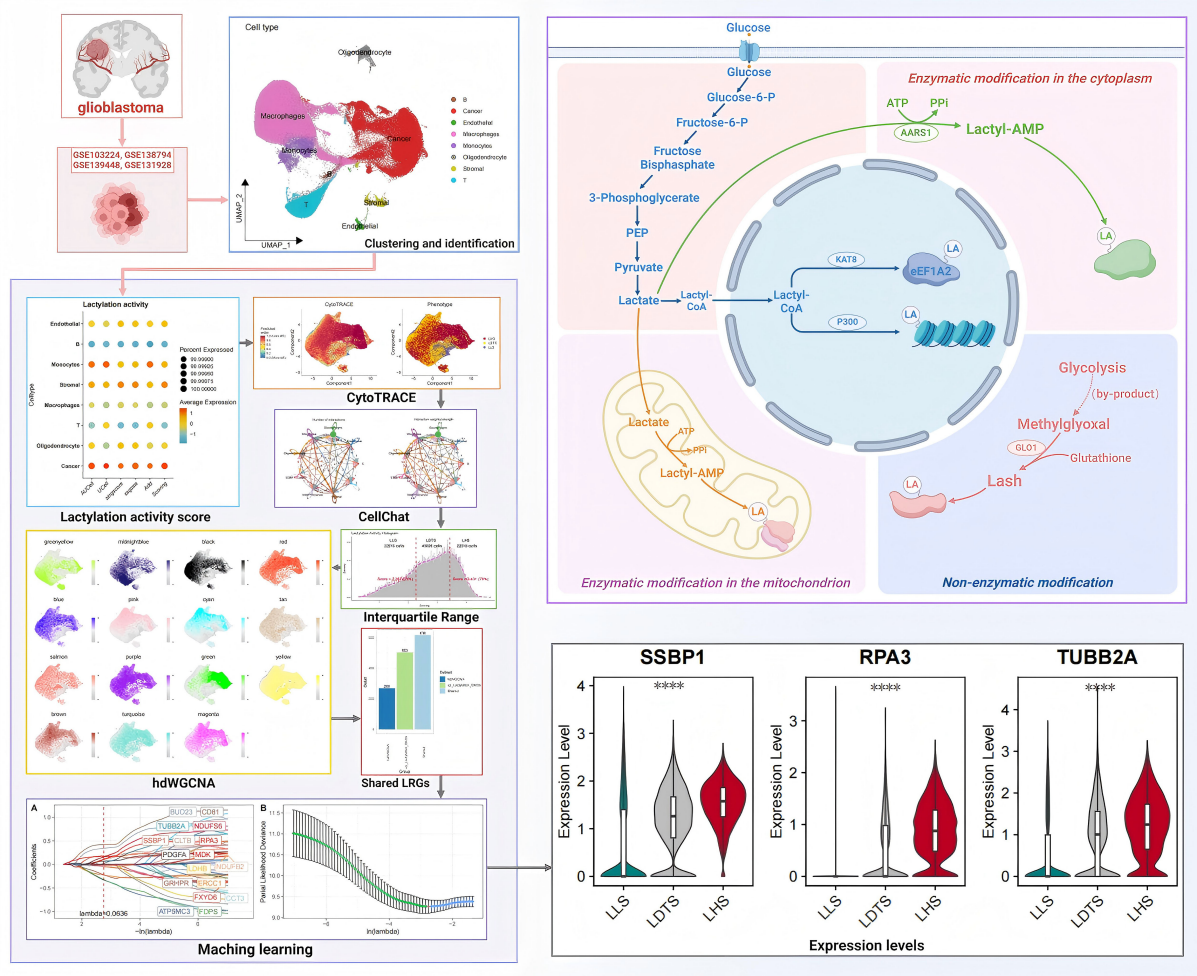
The operational flow chart and mechanism of lactylation. ****P < 0.0001.

In this study, we employed the single-cell RNA sequencing (scRNA-seq) profiles to investigate the heterogeneity in primary and recurrent GBM samples. We applied five machine learning algorithms to explore the relationship between LRGs and GBM progression. Furthermore, we introduced an innovative IQR classification method to re-evaluate the lactylation levels within cancer cell populations at the single-cell level. This approach considers both lactylation and gene expression variations across individual cells. Using 40 independent GBM samples, we demonstrated that the prediction model based on the IQR method exhibits superior robustness and accuracy compared to traditional differential gene expression models and LRGs screening models. The flow chart illustrating the operational procedure and the mechanism of lactylation in this study is presented in **Figure 1**.

## Methods

### Data collection and processing

The scRNA-seq including 40 samples from newly diagnosed GBM (ndGBM, n = 22), recurrent GBM (rGBM, n = 18) were downloaded from GEO database with accession ID: GSE103224, GSE138794, GSE139448 and GSE131928. The bulk RNAseq and microarray data of GBM samples were obtained from TCGA databases. The study incorporated one sample of GBM spatial Transcriptome sequencing (stRNA-seq) with entry number GSE194329. A comprehensive review of previous studies led to the identification and selection of a total of 371 lactylation-related genes (LRGs) (19, 20) (**Supplementary Table 1**).

### scRNA-seq dataset analysis and cell annotation

The “Seurat (Version 4.1.1) (21)” R tool was utilized for data processing to ensure the accuracy of scRNA-seq data. In order to ensure high-quality scRNA-seq data, we screened for genes expressed in a minimum of five individual cells, cells with 200 to 7,000 genes, and cells with more than 20 percent mitochondrial genes. A total of 226736 suitable cells were selected for further investigation. The initial set of highly mutated genes was determined using the “Discover Variant Signatures” function in the “Seurat” package. Principal Component Analysis (PCA) was conducted on highly variable genes to reduce the dimensionality of the scRNA-Seq data by the “RunPCA” function. Following this, the “FindNeighbors” and “FindClusters” functions were applied, resulting in a resolution of 0.8. Cells were annotated based on the expression of marker genes and references to marker gene signatures (**Supplementary Table 2**). we successfully identify and visualize 8 distinct cell clusters using a UMAP plot. The “FindAIIMarkers” tool was utilized for the identification of differentially expressed genes (DEGs) within each cluster. Subsequently, a cut-off threshold and modified criteria of P<0.01 and log2 (Foldchange) >0.25 were employed to ascertain the gene markers for each respective cluster. We evaluate the heterogeneity of cell cycle progression within these clusters by lever lactylation cell cycle markers incorporated in the Seurat package.

### Evaluation of lactylation activity

To calculate lactate activity by evaluating transcription factor activity according to SCENIC’s AUCell method, multiple algorithms were used to reduce the bias that may be introduced by a single algorithm, thus improving the robustness of the results. Subsequently, we normalized the output of each algorithm on a scale of 0 to 1 and performed a comprehensive comparative analysis of all algorithms to derive a final score. The lactylation activity of each cell was evaluated at the individual cellular level, and subsequently, the cumulative lactylation activity was calculated. Based on the median scores obtained, the cells were categorized into groups with high or low lactylation activity. Studies have been conducted to identify genes closely associated with lactylation activity. The “FindMarkers” function was utilized to screen for DEGs and further analyze the results of association analysis and DEGs identification in shared genes. AUcell (Version 1.12.0) (22), Ucell (Version 1.8.0) (23), SingScore (Version 1.0) (24), ssGSEA and AddModuleScore (Version 4.1.1) (25) algorithms were employed in this analytical process. AUCell evaluates the functional status of a cell’s transcriptome by calculating the gene set activity for each cell. Specifically, AUCell employs the area under the cumulative distribution curve (AUC) to measure the expression rank of a given gene set within a single cell. Initially, genes within each cell are ranked based on their expression values, and the activity of the gene set is subsequently quantified using AUC values. This approach demonstrates significant advantages in assessing gene set activity in high-dimensional single-cell data and is particularly effective for evaluating the effects of lowly expressed genes. Similarly, UCell serves as another gene-set activity scoring method but focuses on standardizing the ranking scores of gene expression in single cells. Specifically, UCell assesses the activity of a gene set by calculating the ranking score of each cell within the gene set. These standardized ranking scores reflect the relative expression levels of each cell within the gene set, thereby enabling more precise functional evaluations. The SingScore method evaluates gene set activity by ranking the genes of a given gene set within each cell and calculating the average ranking score for that gene set. ssGSEA compares the expression values of a gene set with the expression levels of other genes in the dataset to compute the relative enrichment score (ES). This score indicates the degree of enrichment of the gene set in a specific cell and facilitates an accurate assessment of the relative activity of the gene set in a single cell. AddModuleScore evaluates the activity level of a gene set by calculating its weighted average expression value in each cell. The computational procedure involves normalizing the gene expression for each cell, calculating the score for the gene set, and normalizing the final score to obtain the module activity score. Through this process, a comprehensive activity score for the gene set in each cell can be achieved.

IQR method serves as a critical tool for classifying the lactoacylation activity of individual cells, thereby addressing the inherent biological heterogeneity. Given the significant variation in lactoacylation levels within tumor populations, this study employed the IQR method to categorize cells into three distinct groups according to their lactoacylation activity. Utilizing the quartile method, cell fractions below the 25th percentile are categorized as the Low lactylation state (LLS) group, those between the 25th and 75th percentiles are classified as the Dynamic transition lactylation state (LDTS) group, and those above the 75th percentile are designated as the High lactylation state (LHS) group. A differential expression analysis was then performed to identify DEGs between the LHS and LLS groups. This analysis led to the identification of 1458 DEGs, which were selected for further investigation (**Supplementary Table 3**).

### The pipeline of high-dimensional WGCNA

To accurately identify highly relevant genes in disease-specific cell subclusters, we employed a computational approach based on high dimensional weighted gene coexpression networks to analyze the scRNA-seq data. Initially, a graph-based clustering algorithm utilizing shared nearest neighbors was utilized for the identification of disease-specific cell subclusters (1). Subsequently, hdWGCNA was applied to the expression data of these cell subclusters to unveil gene modules with strong associations. After acquiring the modular genes, we conducted a differential expression analysis utilizing the FindMarkers function, setting a significance threshold of P<0.05, to identify genes significantly associated with GBM. The intersection of these analyses revealed key genes that play crucial roles in GBM.

### Spatial transcriptomics data analysis

The annotation of cell populations was conducted using hematoxylin and eosin (HE) stained sections along with significantly variable genes within each cluster. The Seurat R package was employed for processing and interpreting spatial transcriptomics (ST) data (26). Standardization of ST data was performed using SCT technology, followed by integration of the data through the functions SelectIntegrationFeatures, PrepSCTIntegration, FindIntegrationAnchors, and IntegrateData. An unsupervised clustering approach was utilized to aggregate similar ST regions. Subsequently, the functions SpatialDimPlot and SpatialFeaturePlot were applied to visualize the expression levels of cells in the ST data. Unique marker genes for each cell type were identified using the Seurat function FindAllMarkers, with a focus on markers exhibiting a positive log2 fold change. Finally, a standard RCTD analysis pipeline was strictly adhered to, concentrating on reference and Visium spatial transcriptomics data in a fully bimodal mode.

### Enrichment analysis

Through the analysis of gene function, high-throughput molecular findings are often translated into practical applications in the field of biology. In this study, we conducted gene function analysis utilizing the “clusterProfiler” (R version 3.18), and visualized the results using Disease Ontology (DO) and Gene Ontology (GO) annotations provided by the Visualization Hub Genes platform.

### Machine learning algorithms are used to identify optimal LRGs

The application of four machine algorithms is employed to identify the optimal LRGs. The first step involves applying the Least Absolute Shrinkage and Selection Operator (LASSO) (27, 28) to iteratively reweight least squares in order to filter candidates. Following 1000 iterations of the algorithm, the feature variables are selected based on the minimum criteria. The candidate genes were identified through a univariate Cox (29) regression analysis, with those exhibiting a p-value less than 0.05 being selected for further consideration. XGboost (30) is an exceptionally effective methodology for addressing a wide array of classification challenges. This approach, which employs extreme gradient boosting, can systematically rank features from most to least important through the utilization of the XGboost package in R. Random forest (RF) (31, 32) offers high predictive accuracy, resilience to overfitting, feature importance evaluation, and applicability to high-dimensional datasets, making it a robust and versatile tool for a wide range of predictive modeling tasks. The Boruta (33) algorithm is a supervised classification feature selection method used to identify all relevant features in a classification problem.

### Pseudotime trajectory, transcription factor analysis and cell-cell interaction analysis

The scRNA data for all cell types in the GBM was analyzed using the R package “Monocle2” (Version 2.18.0) (34) to investigate the correlation between aggregated LRGs and cellular pseudotime tracks. Variant genes that were most representative were selected by estimating cell size factors using the “estimateSizeFactors” function. Pseudo-temporal ordering of cells was performed using the “orderCells” function, and the resulting trajectories were visualized utilizing Monocle’s “plot cell trajectory” function. The R package “CellChat” is utilized for the identification and quantification of cell-to-cell communication between distinct cell types within a single-cell dataset. The “ClusterGVis” software package (version 10.1.0) in conjunction with the k-means clustering algorithm was utilized to visualize the dynamic trends within metabolic pathways.

### Patients and samples

The study protocol was approved by the Ethics Committee of the Affiliated Hospital of Qingdao University (QYFY WZLL 29545). Written informed consent was obtained from each patient before participation in the trial. A total of 4 patients diagnosed with GBM were enrolled, and normal brain tissue adjacent to the tumor was used as control. Tissue samples were immediately frozen in liquid nitrogen and transported to the laboratory for subsequent analysis.

### Quantitative real-time PCR

The RNA was extracted using TRIzol reagent (Bioflux, China). And the extracted RNA (1μg) was reverse-transcribed into cDNA utilizing the PrimeScript™ RT kit (Takara Biomedical Technology Corporation, Beijing, China). RT-qPCR analysis was conducted on CFX Opus instrument (Bio-Rad Laboratories Corporation, Shanghai, China) employing Talent qPCR PreMix (SYBR Green). The entire reaction followed a thermal cycle procedure consisting of 50 cycles with 10 seconds at 95°C and 20 seconds at 60°C. The relative quantification method used for data analysis was the 2−ΔΔCt method. The primer sequences are shown in **Supplementary Table 4**.

### Western blot

RIPA lysis buffer (Solarbio, China) was utilized to lyse the tissues. Protein concentrations were quantified using the BCA method. Samples were subjected to electrophoresis on a 10% sodium dodecyl sulfate polyacrylamide gel and subsequently transferred onto PVDF membranes. Non-specific binding sites on the membranes were blocked with protein-free rapid blocking buffer (Meilun, Dalian, China) for 20 minutes at room temperature. The membranes were then incubated overnight at 4°C with primary antibodies against TUBB2A, RPA3, SSBP1, and β-actin, all sourced from China Protein Corporation. Subsequently, the membranes were incubated with goat anti-rabbit IgG for 1 hours at room temperature. The protein bands were then visualized using a chemiluminescence imaging system (Millipore, Billerica, MA, USA). (Solarbio, China), with β-actin serving as a loading control. Western blot analysis was employed to assess the protein levels of SSBP1, RPA3, and TUBB2A in both the normal and GBM groups. Relative protein expression levels were quantified based on the intensity of the corresponding bands.

### Immunoprecipitation

Cell lysates, supplemented with protease inhibitors, were incubated with anti-TUBB2A antibody (Proteintech, China) and magnetic beads for 12 hours at 4°C. The magnetic beads were subsequently washed three times with lysis buffer to ensure thorough removal of non-specifically bound proteins. Finally, the proteins specifically bound to the magnetic beads were eluted and prepared for Western blot analysis.

### Statistical analysis

Logistic regression analysis was conducted utilizing the GLM function to examine the association between variables and binary outcomes. Continuous variables were compared through the Wilcoxon rank-sum test. Kaplan-Meier survival analyses were performed, with significance assessed using the log-rank test. Receiver Operating Characteristic (ROC) curves were constructed using the “TimeROC” package, while calibration curves were generated using the “RMS” package. A p-value of less than 0.05 was considered statistically significant.

## Results

### The characteristics of GBM lactylation were analyzed using the scRNA-seq dataset

**Figure 2:**
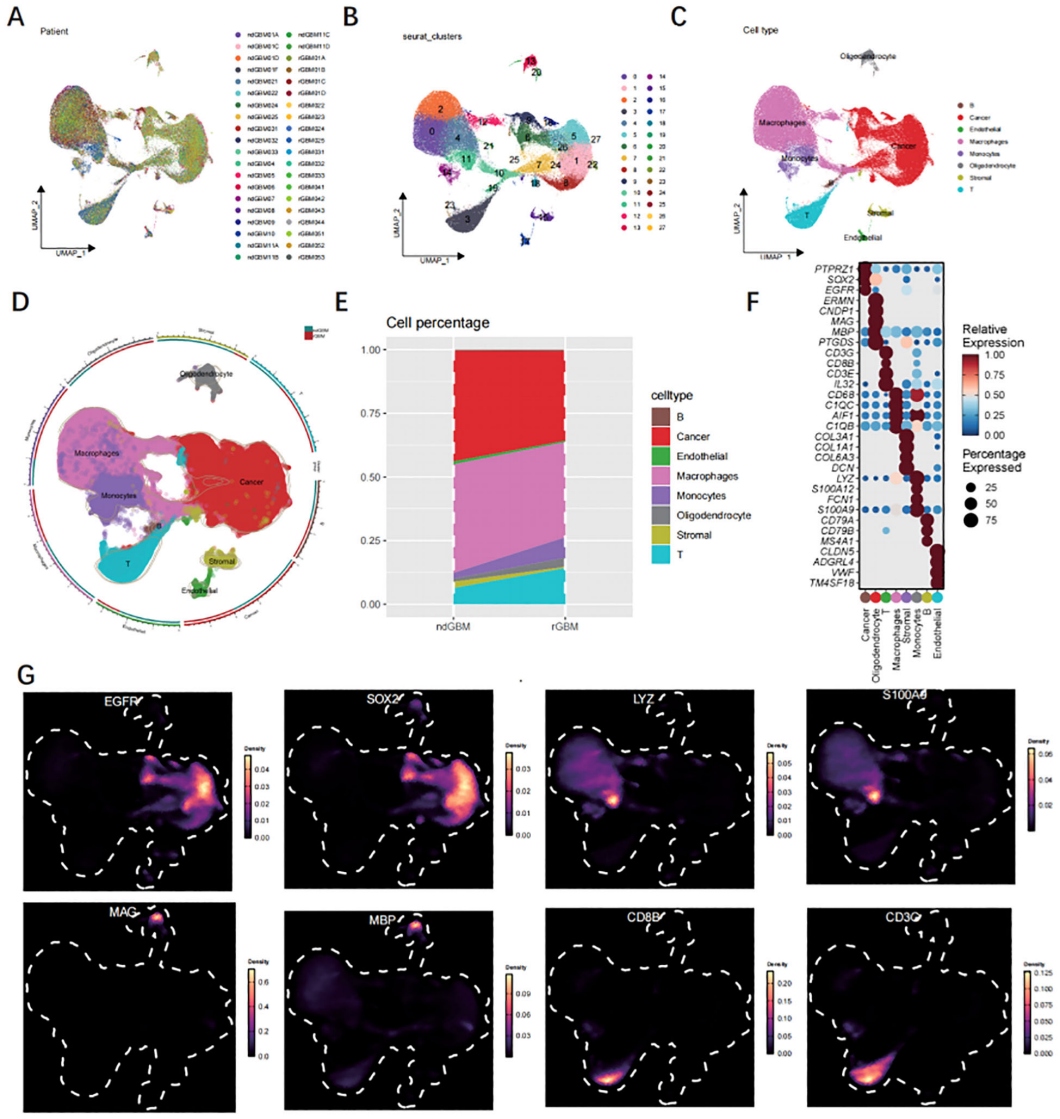
Analysis of single-cell data. **(A)** The UMAP profiles of 40 samples. **(B)** The UMAP diagram is colored according to the 18 cell clusters. **(C, D)** The cell type annotations are clustered in the scRNA-seq data using seurat UMAP. **(E)** The cellular composition of GBM and normal tissues. **(F)** Bubble map illustrating marker genes associated with 11 major cell types. **(G)** The UMAP visualization depicts the expression patterns of 9 genes exhibiting high levels of expression.

The heterogeneity of GBM was investigated by extracting three samples from the GEO database (**Figure 2A**), and single-cell sequencing data was utilized to assess differences between ndGBM samples and rGBM samples. Following the identification of 2,000 highly variable genes, PCA was employed for dimensionality reduction, with a focus on the top 20 principal components (PCs). Subsequently, 27 clusters were generated (**Figure 2B**). Manual annotation based on classical marker genes revealed the presence of 8 distinct cell types (**Figures 2C, D**). **Figure 2E** illustrates alterations in the distribution of cellular proportions. The expression patterns of signature marker genes associated with 8 cell subpopulations are depicted in **Figure 2F** Bubble maps. Cell type recognition relies on the marker genes illustrated in **Figure 2G**.

**Figure 3:**
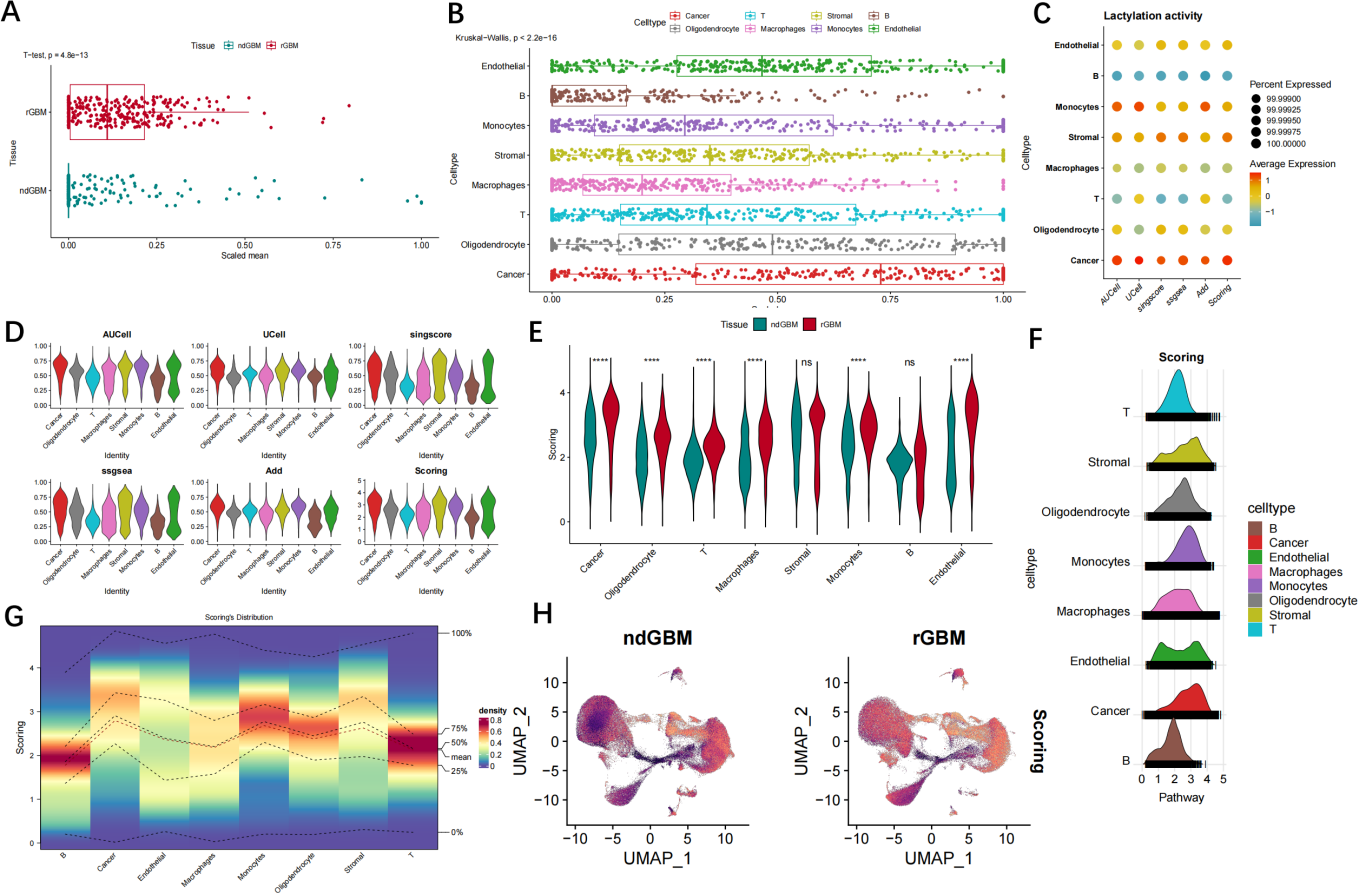
The analysis of lactylation activity in individual cells among patients with GBM. **(A)** The Wilcoxon tests were conducted to compare lactylation-related gene sets between normal individuals and patients with GBM. **(B, C)** The bubble map displays the enrichment scores of genes associated with lactylation for each individual cell type within the GBM. **(D)** The results from the analysis of five algorithms indicated that cancer cell and stromal cell exhibited the highest level of lactylation activity, whereas B cells and neutrophils demonstrated relatively lower levels of lactylation activity. **(E–G)** The lactylation activity levels differ among cell types in both normal and GBM samples. **(H)** The UMAP plot illustrates the lactylation scores for diverse cell subsets. ns, no significance; ****P < 0.0001.

Next, we analyzed the lactylation activity in the samples, and the GSVA analysis results showed that the rGBM samples showed higher lactylation activity compared with the ndGBM group (**Figure 3A**). The lactylation activity of each cell was calculated using AUcell, Ucell, SingScore, ssGSEA, and AddModuleScore algorithms. The results revealed heterogeneity in the lactylation activity of GBM cell clusters compared to the control group. Cancer cell, monocytes and stromal cell exhibited the highest agglutination activity, whereas T cell, neutrophil, and B cell displayed relatively low activity (**Figures 3B–D**). The lactylation activity levels between GBM and control samples in each cell type exhibited significant disparities, with notable distinctions observed in cancer cell, monocytes and stromal cell (**Figures 3E–G**). **Figure 3H** illustrates the lactylation scores for diverse cell subsets.

### Visualization of lactylation activity and heterogeneity in cancer cell clusters

**Figure 4:**
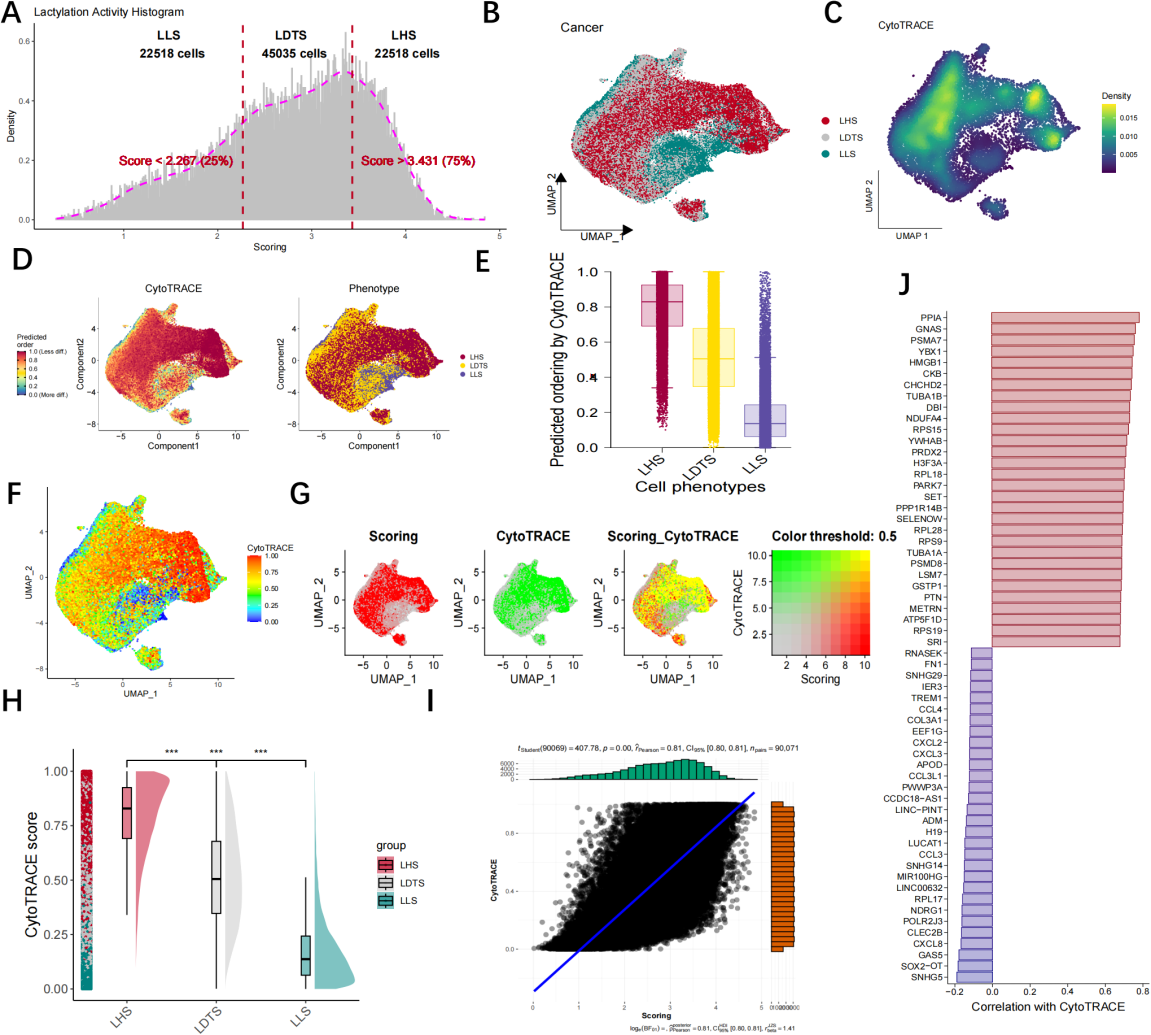
Lactylation activity and heterogeneity were visualized by CytoTRACE. **(A)** The DEGs were categorized into LHS, LDTS, LLS group based on the IQR method. **(B, C)** The UMAP map illustrating the cell distribution of LHS, LDTS and LLS. **(D)** The CytoTRACE characteristics and lactylation-related phenotypes of GBM cancer cells. **(E)** Boxplots showing differentiating ordering identically ordered by CytoTRACE. **(F–G)** The correlation was revealed when the lactylation score was combined with the CytoTRACE score. **(H)** Comparison of CytoTRACE Scores Among LHS, LDTS and LLS. **(I)** Pearson correlation test for CytoTRACE and lactylation scores **(J)** The expression Profiles of Cell-Specific Marker Genes Linked to CytoTRACE Analysis. ***P < 0.001.

The AUCell R package was utilized to measure the lactylation activity of each cell, with higher AUC values indicating greater lactylation activity, we computed the corresponding lactylation AUC score for each cell and categorized them into LHS, LDTS and LLS AUC groups based on the IQR range methods (**Figure 4A**). Cell Counting and Expressed Gene Trace Reconstruction Analysis (CytoTRACE) is a computational methodology designed to infer the relative differentiation status of cells based on single-cell RNA sequencing data. In this study, we examined the differentiation status of three cell groups (LHS, LDTS and LLS) utilizing the CytoTRACE algorithm. **Figures 4B, C** illustrate the distribution and density of cancer cells across groups LHS, LDTS and LLS. The differentiation status of cancer cells was further investigated using the Monocle2 algorithm through CytoTRACE analysis. The results demonstrated that LHS cancer cells exhibited a lower degree of differentiation and possessed a higher potential for differentiation (**Figures 4D, E**). We subsequently correlated the lactylation score with the CytoTRACE score, as illustrated in **Figures 4F, G**. The yellow regions indicate cells that exhibit high lactylation and CytoTRACE scores, which largely demonstrate a consistent overlap. We then conducted an analysis of the CytoTRACE scores across the three groups (**Figure 4H**). Additionally, our findings revealed a significant correlation between CytoTRACE and lactylation scores (**Figures 4I**). **Figure 4J** illustrates the expression profiles of cell-specific marker genes in relation to CytoTRACE.

### HdWGCNA identifies hub genes associated with cancer cell

**Figure 5:**
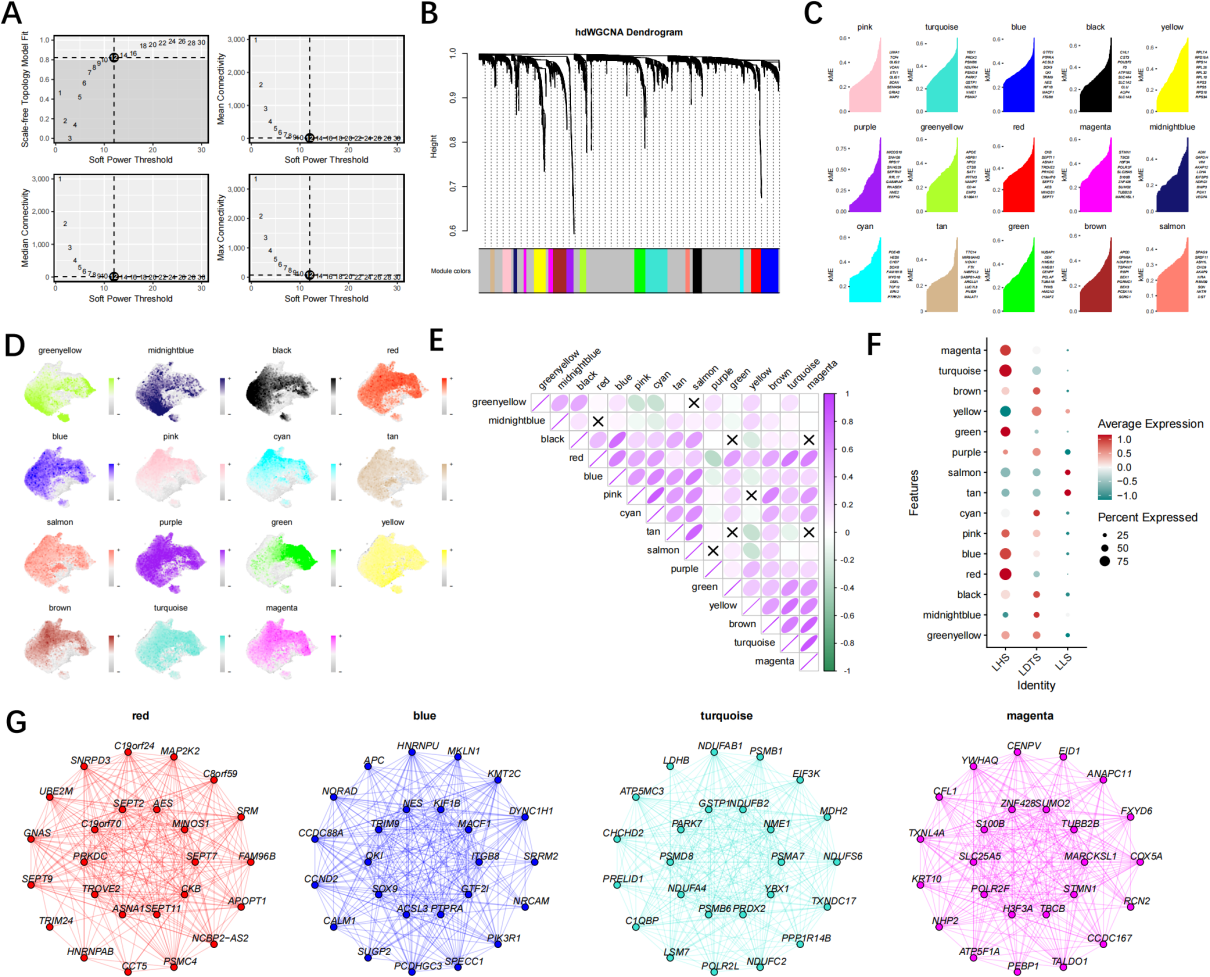
The hdWGCNA analysis reveals that the red, turquoise, blue, and magenta modules are the four hub modules closely associated with high-lactylation group. **(A)** Selecting a soft power of 12 to construct a scale-free network. **(B)** Visualizing eight modules in a scale-free network using a tree diagram. **(C)** Obtaining 15 gene modules and presenting the top hub gene based on the hdWGCNA pipeline. **(D)** Examining the distribution of 15 gene modules in both LHS, LDTS and LLS groups. **(E)** The correlation between gene modules. **(F)** The degree of expression of gene modules in groups LHS, LDTS and LLS. **(G)** Creating a correlation bubble diagram to illustrate module associations into the LHS, LDTS and LLS group.

The hdWGCNA algorithm is utilized to identify key modules exhibiting cancer cell characteristics. A scale-free fibroblast network, based on optimal connectivity, is constructed using a soft threshold of 12. Consequently, a total of 15 gene modules are identified (**Figures 5A–D**). The correlation analysis between these 15 gene modules and among the gene modules and the LHS, LDTS and LLS groups facilitated the identification of gene modules strongly associated with the LHS, LDTS and LLS groups. The findings indicated that the red, turquoise, blue, and magenta module exhibited the highest level of expression in the LHS group. (**Figures 5E, F**). Notably, All four modules exhibited significant positive correlations with cancer cells. The central genes for subsequent analysis were selected as the top 25 KME genes from the black, turquoise, blue, and brown modules (**Figure 5G**).

### The enrichment analysis and spatial transcriptome analysis

**Figure 6:**
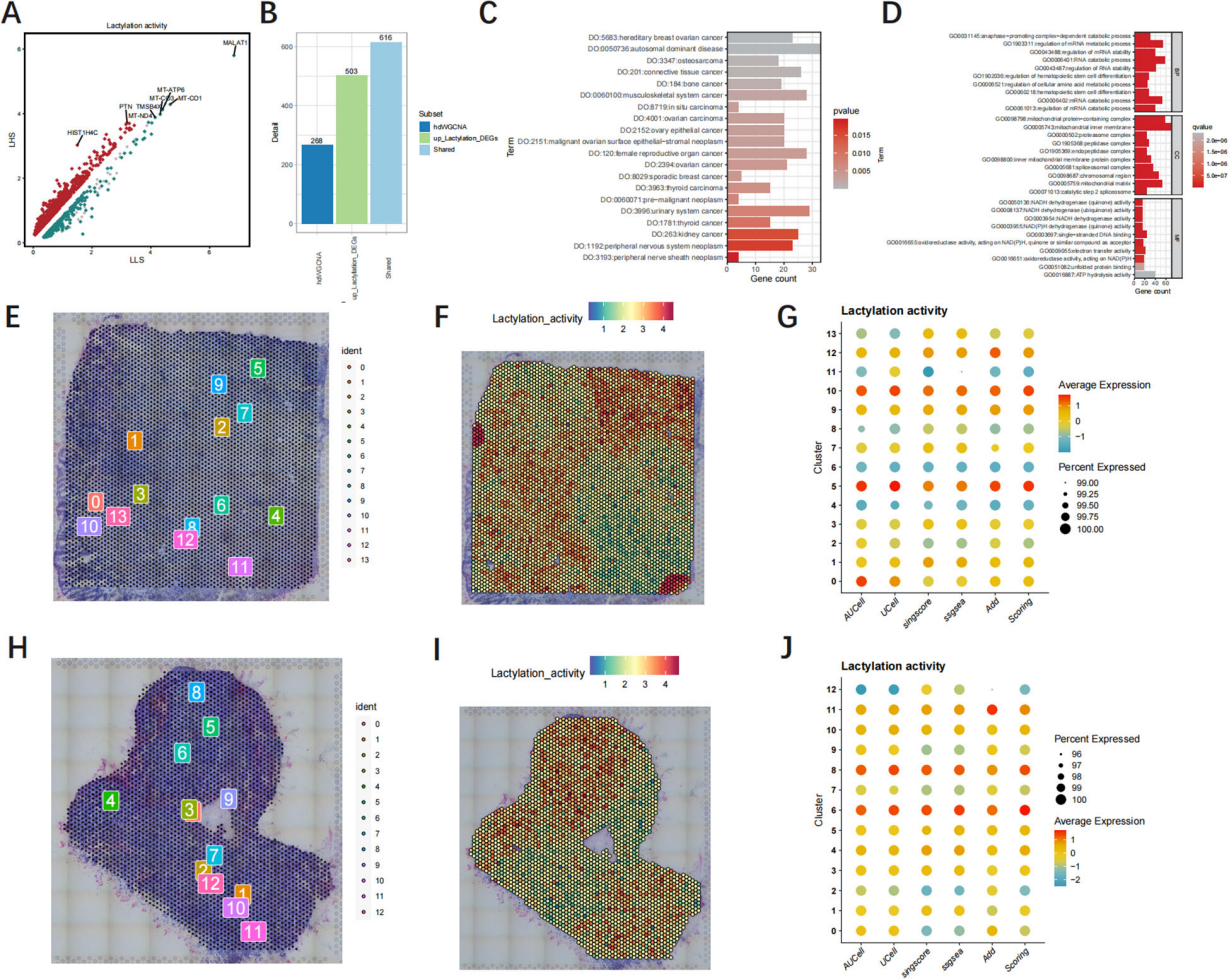
The functional enrichment analysis and Spatial transcriptome analysis. **(A)** The volcanic map of the DEGs. **(B)** Overlap genes in hdWGCNA and DEGs. **(C, D)** GO and DO functional enrichment analysis of shared LRGs. **(E, H)** Cell counts in tissue samples. **(F, I)** The spatial distribution map of lactylation intensity. **(G, J)** The dot plot illustrates the lactylation activity of each population of cells.

The volcano plot shows 1458 DEGs of LHS and LLS (**Figure 6A**). The analysis identified a total of 616 genes that were found in both hdWGCNA and DEGs, indicating their involvement in up-regulating lactylation activity (**Figure 6B**). The consequences of DO analysis illustrate that shared LRGs are relevant to connective tissue cancer, autosomal dominant disease and ovary epithelial cancer (**Figure 6C**, **Supplementary Table 5**). The GO analysis revealed that shared LRGs were significantly enriched in anaphase−promoting complex−dependent catabolic process, regulation of mRNA metabolic process and actin binding (**Figure 6D**, **Supplementary Table 6**). We conducted an in-depth analysis of the spatial architecture of GBM samples. Thereafter, we delineated cancerous regions based on HE staining (**Figures 6E, H**). Notably, the red-stained areas represent cancer cells with elevated lactylation levels (**Figures 6F, I**). Additionally, The dot plot illustrates the lactylation activity of each population of cells (**Figures 6G, J**).

### Identification and validation of hub LRGs by machine learning

**Figure 7:**
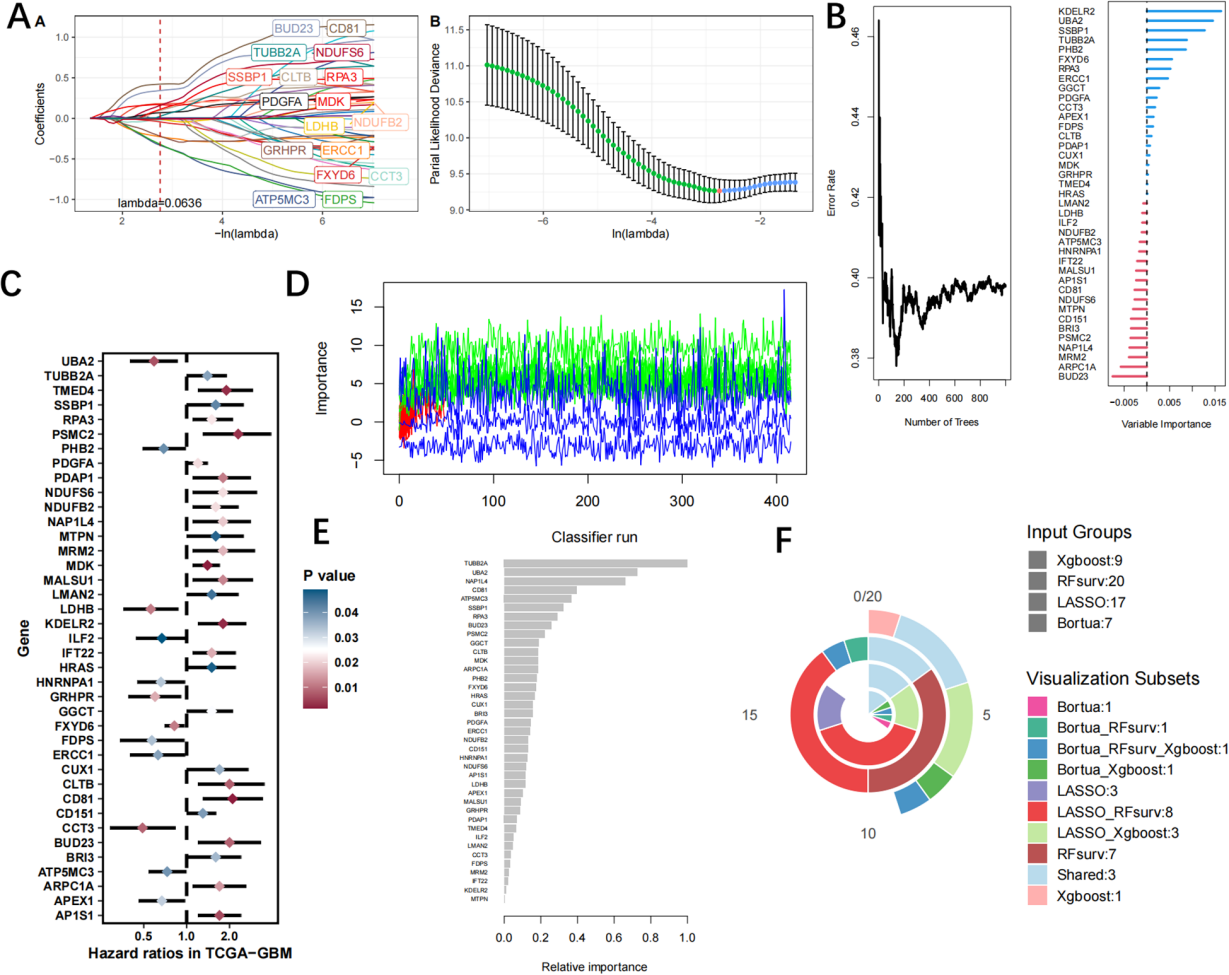
The identification of hub LRGs is performed using machine learning techniques, **(A)** the LASSO regression algorithm, **(B)** RF algorithm. **(C)** Ucox algorithm. **(D)** Boruta algorithm. **(E)** xgboost algorithm. **(F)** Venn diagrams of four algorithms.

We utilized machine learning algorithms to further refine the selection of hub LRGs with significant diagnostic value. A total of 17 genes were identified through LASSO analysis (**Figure 7A**, **Supplementary Table 7**). Subsequently, we identified 20 hub genes using the RF algorithm (**Figure 7B**, **Supplementary Table 8**). And an additional 39 hub genes were identified using the Uncox (**Figure 7C**, **Supplementary Table 9**) and 7 hub genes of the Boruta algorithm (**Figure 7D**, **Supplementary Table 10**). Furthermore, the xgboost algorithm was employed to select another set of 9 hub genes (**Figure 7E**, **Supplementary Table 11**). Finally, by performing intersection analysis on the signature genes identified through these five machine learning algorithms, we determined three common hub genes: SSBP1, RPA3, and TUBB2A (**Figure 7F**).

### Reveal the hub LRGs at the single-cell level

**Figure 8:**
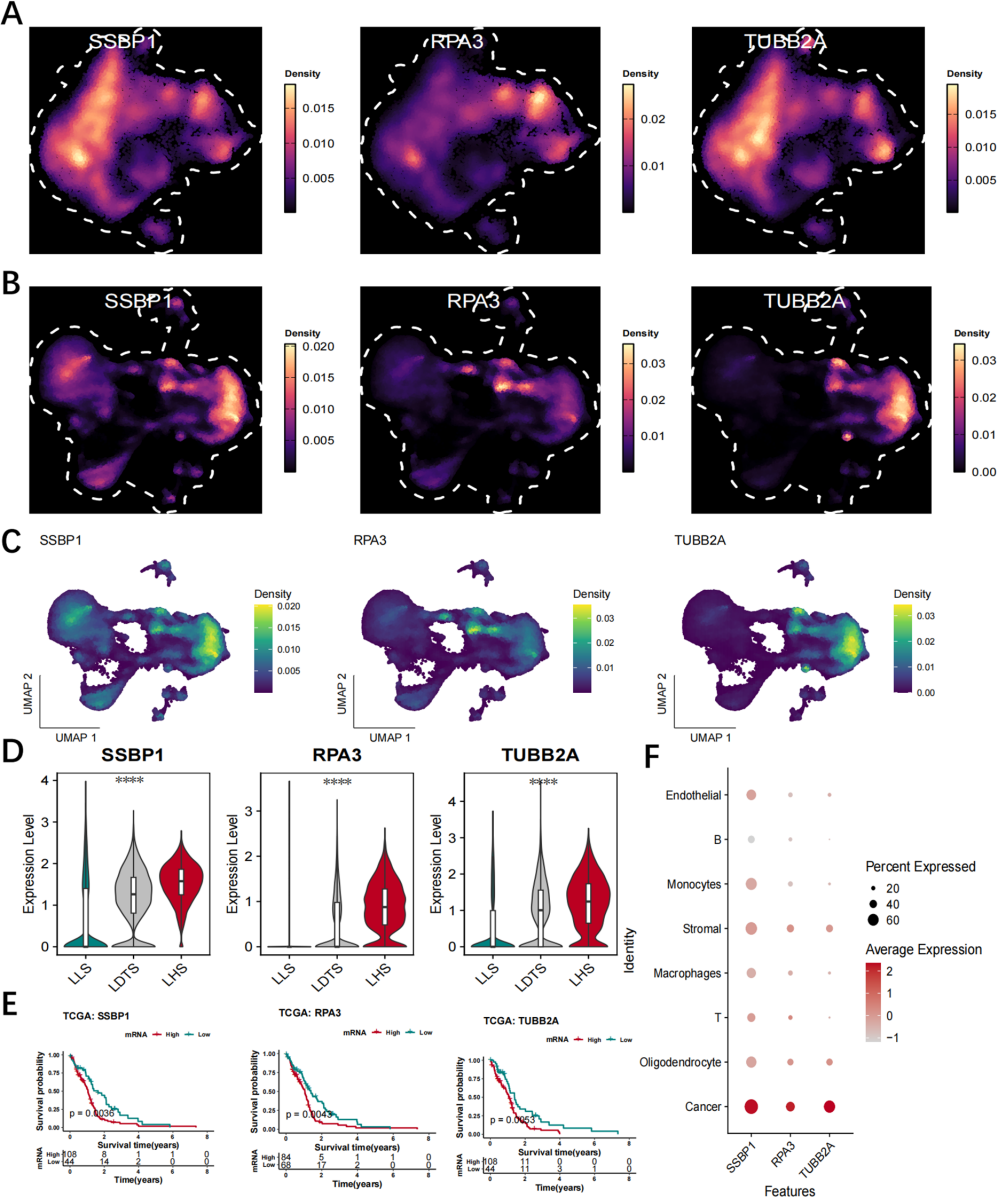
Validate hub LRGs at the single-cell level. **(A)** UMAP analysis revealed that these genes were predominantly expressed in LHS. **(B, C)** UMAP analysis revealed that these genes were predominantly expressed in cancer cells. **(D, E)** The expression levels of SSBP1, RPA3, and TUBB2A were observed in LHS, LDTS and LLS. **(F)** The analysis of the expression profiles of SSBP1, RPA3 and TUBB2A across diverse cellular types. ****P < 0.0001.

The UMAP analysis demonstrated that these LRGs were predominantly expressed in cancer cells and LHS cells (**Figures 8A–C**). **Figures 8D, E** illustrates the differential expression levels of SSBP1, RPA3 and TUBB2A between the LHS, LDTS and LLS. The results demonstrated that SSBP1, RPA3 and TUBB2A exhibited high expression levels in cancer cells compared to relatively lower levels in T cells and B cells (**Figure 8F**).

### Results of trajectory analysis and cell interactions

**Figure 9:**
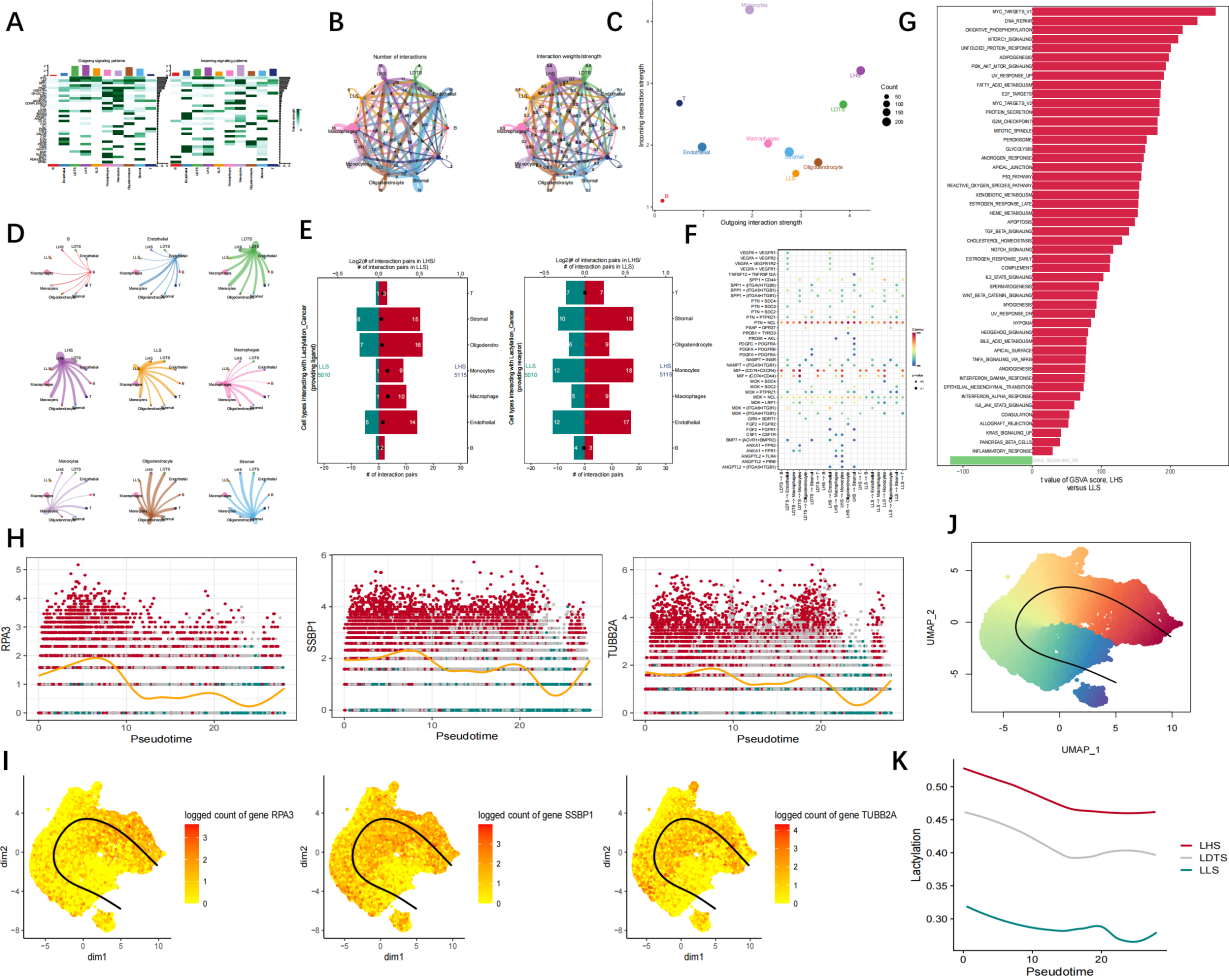
Cell trajectory and communication analysis. **(A)** The interaction quantity and weight/strength of cellular interactions within the communication network of GBM. **(B–D)** The results of cell communication demonstrated the quantification and intensity of intercellular communication between LHS, LLS, LDTS as well as other cellular subtypes. **(E)** Number of cell interaction pairs between LHS and LLS and various cell types. **(F)** The interaction quantity and strength of cell interactions in the communication network of GBM. **(G)** GSVA analysis of LHS. **(H, I)** Pseudotime analysis reflects the expression level of key genes in VSMC. **(J)** Pseudotime UMAP map. **(K)** Relationship between lactylation and cell trajectories.

The findings of cellular communication suggest that LHS exhibits a higher propensity for signaling to neighboring cells via VEGF, BMP, and also demonstrates an increased receptivity towards signals transmitted through SPP1, PTN pathways (**Figure 9A**). The results of cell communication revealed a significantly high frequency of interaction between LHS and LDTS LHS and stromal, as well as monocyrtes and LHS (**Figures 9B–D**). Subsequently, we conducted an analysis of the number of ligand-receptor pairs involving LHS and LLS with various cell types. The results indicated that LHS exhibited the highest number of pairs with oligodendrocytes, stromal cells, and monocytes, respectively. Additionally, LLS demonstrated the highest number of pairs with stromal cells, endothelial cells, and monocytes (**Figure 9E**). The results demonstrated that cancer cells interact with stromal cells via the PTN-NCL and MIF-(CD74+CXCR4) signaling pathways. (**Figure 9F**). GSVA analysis showed that LHS was strongly correlated with OXIDATIVE PHOSPHORYLATION, PI3K AKT MTOR SIGNALING, DNA REPAIR (**Figure 9G**). We divided cancer cells into six groups: SSBP1+ cancer group, SSBP1− cancer group, RPA3+ cancer group and RPA3− cancer group, TUBB2A+ cancer group and TUBB2A− cancer group. Monocle2 was used for trajectory analysis to explore transcriptional heterogeneity in cancer cells. The proportion of SSBP1, RPA3, and TUBB2A cancer cells increased synchronously during the quasi-time course (**Figures 9H, I**). The pseudo-time series analysis was employed to elucidate the developmental trajectory of cells within each population. The cells exhibiting similar states are clustered together, while distinct cell clusters are visually distinguished using color codes, the differentiation trajectory is represented by a black line (**Figure 9J**). Pseudotime analysis revealed a gradual decline in LHS, LDTS and LLS over time (**Figure 9K**).

### Experimental validation of hub LRGs

**Figure 10:**
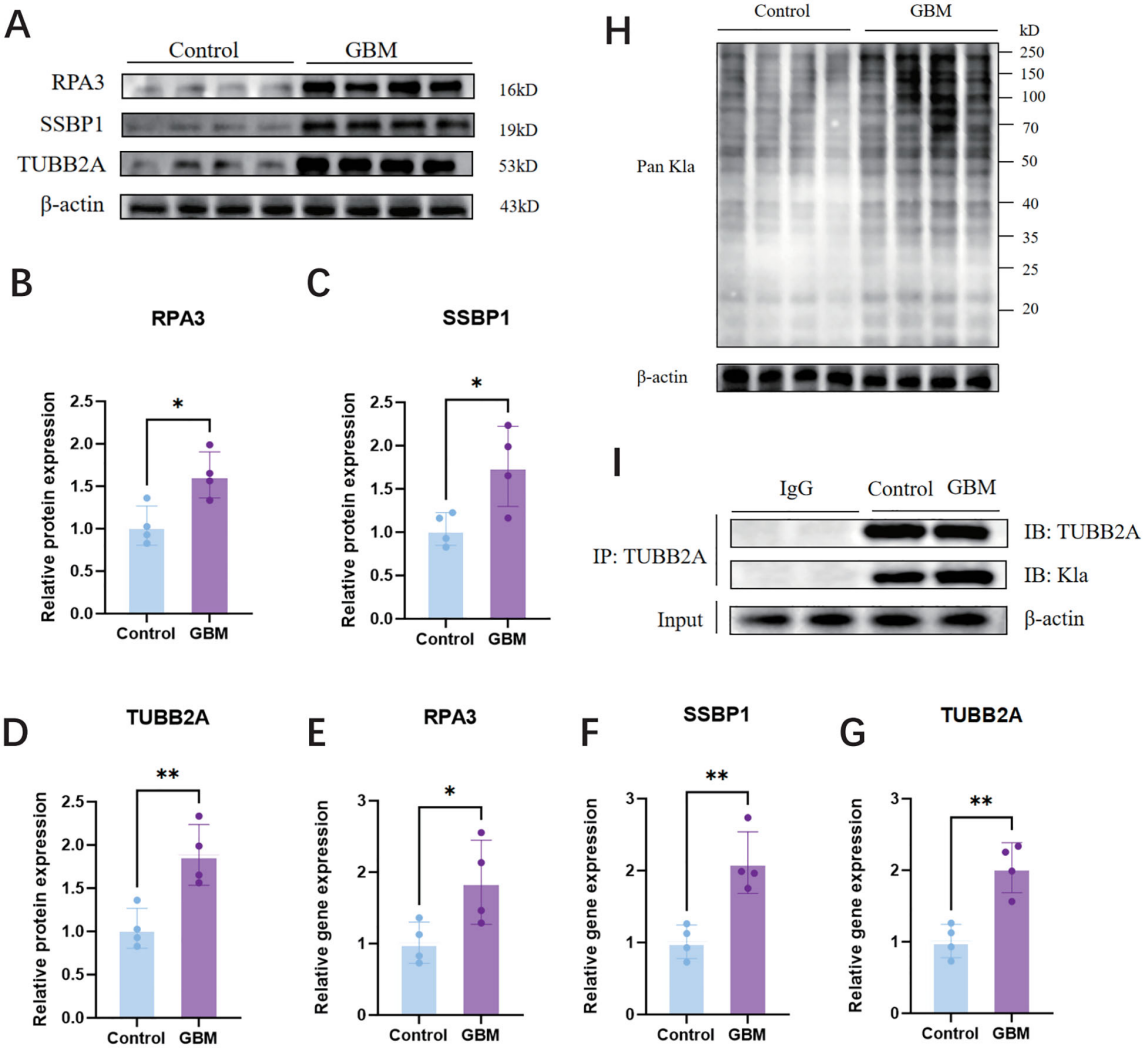
Expression of SSBPP1,RPA3 and TUBB2A in human GBM tissues and control tissues, IP validation of TUBB2A and validation of pan-Kla. **(A)** WB analysis of SSBPP1, RPA3, and TUBB2A in GBM tissues and control tissues. **(B–D)** Quantitative analysis of **(A)**; n = 4 per group. **(E–G)** RT-qPCR analysis of SSBPP1,RPA3 and TUBB2A mRNA levels in human GBM and control tissues; n = 4 in per group. **(H)** The level of lactylation was measured by Western blot in human GBM tissues and control tissues. **(I)** IP validation of TUBB2A. *P < 0.05; **P < 0.01.

Firstly, we observed a significant increase in the protein levels of SSBPP1, RPA3, and TUBB2A as shown in **Figures 10A–D**, as well as an elevation in their mRNA levels as demonstrated in **Figures 10E–G**, using WB and RT-qPCR analyses. We subsequently investigated the global lactylation levels in human GBM tissues compared to control tissues. The results demonstrated that GBM tissues exhibited significantly elevated lactylation levels relative to control tissues (**Figure 10H**). Finally, we selected TUBB2A with the most significant difference for IP validation, and WB results showed that TUBB2A was involved in the lactylation modification process in GBM and was significantly stronger than the control group (**Figure 10I**).

## Discussion

Aberrant reprogramming of tumor metabolism represents a hallmark of cancer development, with lactic acid production being a pivotal process in this metabolic shift. Cancer cells exhibit a propensity to generate lactate through glycolysis even in the presence of ample oxygen. The accumulation of lactic acid not only fulfills the demands of rapid cancer cell proliferation but also facilitates tumor growth, invasion, immune evasion, and therapeutic resistance by modulating the tumor microenvironment. Previous transcriptomics studies in GBM have primarily concentrated on identifying prognostic genes and predicting clinical outcomes (35). However, these studies frequently neglect the intricate landscape of intercellular heterogeneity. Meanwhile, scRNA-seq studies on GBM predominantly focus on elucidating the functions of various cellular components, discovering novel cell subsets, and investigating cell-to-cell heterogeneity. Nevertheless, due to limited sample sizes, there is a paucity of analyses linking specific cancer cell subsets to patient prognosis. Moreover, most existing models are constrained by biases stemming from reliance on absolute gene expression values. These models typically treat cell subsets as homogeneous entities, thereby overlooking the differential expression patterns of mechanisms such as lactate metabolism, senescence, and oxidative stress within individual cells. Such oversimplified modeling approaches often significantly compromise the accuracy of their predictions, thereby impeding their practical utility.

We initially used scRNA-seq data to identify a total of eight different cell subsets, including cancer cells and macrophages. Furthermore, we employed algorithms such as AUCell and UCell to calculate the lactylation score of GBM, We performed a 0 to one normalization for each algorithm and a comprehensive comparative analysis of all algorithms to arrive at the final score, revealing that cancer cells exhibited the highest lactylation score. From a biological standpoint, the tumor microenvironment is marked by substantial metabolic reprogramming. Within this environment, cells exhibit diverse metabolic profiles, including varying levels of lactylation production. Cancer cells, in particular, undergo rapid and heterogeneous metabolic adaptations to sustain their accelerated proliferation and survival under metabolically stressful conditions. In GBM, increased lactate production via aerobic glycolysis leads to considerable variation in lactylation modification among individual cells, reflecting the dynamic metabolic state of these cells. By categorizing cells into low, intermediate, and high lactylation-producing groups using IQR analysis, we effectively captured the inherent biological heterogeneity. This approach enables the identification of distinct cell subpopulations that may differentially contribute to tumor. Furthermore, lactylation enhances the resistance of cancer cells to radiotherapy and chemotherapy by modulating signaling pathways such as STAT3 (36). Lactylation also promotes cancer cell invasion and metastasis through the regulation of genes involved in extracellular matrix remodeling. Research has demonstrated that proteins modified by lactylation play a crucial role in regulating cancer cell adhesion, migration, and invasion (37).

To investigate the spatial distribution of these cell subsets within the tumor microenvironment and differentiate gene expression patterns across various regions, we utilized spatial transcriptomics. Through hdWGCNA and machine learning analyses, we identified SSBP1, RPA3, and TUBB2A. Located on human chromosome 6, TUBB2A is the gene that encodes the β-2A tubulin subunit (38), a critical component for the formation of microtubules. Microtubules play an essential role in various cellular processes such as cell division, intracellular transport, and the maintenance of cell morphology (39). Wang et al. (40). demonstrated that TUBB2A may play a crucial role in caspase-dependent apoptosis in glioma cells and could be implicated in the regulation of metabolism-related functions and pathways. Mitochondrial SSBP1 is a housekeeping gene essential for mitochondrial biogenesis through its role in maintaining mitochondrial genome stability (41, 42). As a critical subunit of the SSB complex, SSBP1 plays a pivotal role in regulating various important cellular physiological processes, including the maintenance of mitochondrial DNA content and modulation of metabolic status (42). Su et al. (43). further revealed through bioinformatics analysis that SSBP1 was significantly upregulated in GBM. Their study also indicated that the knockdown of SSBP1 resulted in impaired GBM cell growth and migration, as well as mitochondrial dysfunction. RPA3 plays a critical role in regulating DNA replication. Aberrant expression of RPA3 has been associated with genomic instability and the onset and progression of various tumors (44). A recent study (45) investigated the relationship between RPA3 and immune cell infiltration and activation by constructing a univariate Cox regression model to predict the prognosis of glioma patients. The findings indicated that overexpression of RPA3 enhances the proliferation, migration, and invasion of glioma cells through the phosphorylation of PI3K, AKT, and mTOR, thereby activating the PI3K-AKT-mTOR signaling pathway (46).

We attach significant importance to the biological implications underlying our findings. Upon applying the IQR scoring framework to the scRNA-seq data, we observed distinct differences among cancer cells from LHS, LDTS, and LLS. Specifically, in terms of cell communication analysis, it was noted that cancer cells from LHS exhibit distinct interactions with immune cells, significantly contributing to tumor progression and metastasis. Given that tumor tissues encompass cells at various developmental stages, we conducted an investigation into the relationship between LHS and the developmental trajectories of cancer cells through pseudo-temporal analysis. Our results indicate that cells from LHS are more likely to possess characteristics of cancer stem cells, whereas cells from LLS are closer to the terminal stages of differentiation. Similarly, we found that score-related genes exhibit substantial variation across different stages of cancer cell progression. These observations suggest that the IQR scoring framework has a higher capacity to influence the differentiation of cancer cells. Furthermore, by integrating the IQR scoring framework into the deconvoluted GBM spatial transcriptome, we observed variations in IQR scores among tumors, indicating differing degrees of lactylation in cancer cells. Intercellular communication analysis highlights the critical role of the PTN-NCL pathway. The multifunctional protein nucleolin (NCL) is overexpressed on the surface of activated endothelial cells and tumor cells, mediating the stimulatory effects of various angiogenic growth factors. For instance, pleiotropic cell growth factor (PTN) induces its effects through both NCL and αvβ3 integrin, which is essential for PTN-induced cell migration. A prior study (47) demonstrated a positive correlation between cell surface NCL and αvβ3 expression in human glioblastoma tissue arrays. Furthermore, the inhibition of cell migration by a cell surface NCL antagonist was observed exclusively in cells expressing αvβ3, thereby validating the rationale of the PTN-NCL pathway.

The analysis conducted as a pilot study had certain notable limitations, including the relatively low sequencing depth of the scRNA-seq data and the limited sample size. Consequently, it is imperative to conduct further validation in a larger cohort of patients to substantiate our conclusions. Furthermore, Given the relatively modest sample size employed in our experimental validation, we recognize that the results of our Western blot and RT-qPCR analyses should be interpreted with due caution. The limited sample size constrains the statistical power of these experiments, and additional validation using a larger cohort of GBM patients will be critical to confirm the robustness of our findings. While the data from this pilot study are promising, more extensive studies are necessary to comprehensively evaluate the reliability and generalizability of the biomarkers associated with lactylation identified herein. And additional research is required to clarify the regulatory relationships and underlying mechanisms of SSBP1, RPA3 and TUBB2A in Cancer cell lactylation despite demonstrating an association between them. The present study establishes a genetic signature linked to the process of lactylation, which can serve as a diagnostic and prognostic indicator for immune-related adverse events. This characteristic offers a potential clinical tool to gain novel insights into patient prognosis and response to immune checkpoint blockade therapy.

## Data Availability

The original contributions presented in the study are included in the article/**Supplementary Material**. Further inquiries can be directed to the corresponding authors.
